# Methanolic extract of white asparagus shoots activates TRAIL apoptotic death pathway in human cancer cells and inhibits colon carcinogenesis in a preclinical model

**DOI:** 10.3892/ijo.2013.1976

**Published:** 2013-06-07

**Authors:** SOUAD BOUSSEROUEL, JULIE LE GRANDOIS, FRANCINE GOSSÉ, DALAL WERNER, STEPHAN W. BARTH, ERIC MARCHIONI, JACQUES MARESCAUX, FRANCIS RAUL

**Affiliations:** 1 University of Strasbourg, Unit EA 4438, Faculty of Medicine;; 2 Institut de Recherche contre les Cancers de l’Appareil Digestif (IRCAD), Strasbourg;; 3 AERIAL, Illkirch, France;; 4 Max Rubner Institute, Karlsruhe, Germany;; 5 University of Strasbourg, Faculty of Pharmacy, IPHC, CNRS-UMR7178, Illkirch, France

**Keywords:** *Asparagus officinalis*, colorectal cancer, SW480 cells, SW620 cells, aberrant crypt foci, inflammation, caspases, death receptors

## Abstract

Shoots of white asparagus are a popular vegetable dish, known to be rich in many bioactive phytochemicals reported to possess antioxidant, and anti-inflammatory and antitumor activities. We evaluated the anticancer mechanisms of a methanolic extract of *Asparagus officinalis* L. shoots (Asp) on human colon carcinoma cells (SW480) and their derived metastatic cells (SW620), and Asp chemopreventive properties were also assessed in a model of colon carcinogenesis. SW480 and SW620 cell proliferation was inhibited by 80% after exposure to Asp (80 *μ*g/ml). We demonstrated that Asp induced cell death through the activation of TRAIL DR4/DR5 death receptors leading to the activation of caspase-8 and caspase-3 and to cell apoptosis. By specific blocking agents of DR4/DR5 receptors we were able to prevent Asp-triggered cell death confirming the key role of DR4/DR5 receptors. We found also that Asp (80 *μ*g/ml) was able to potentiate the effects of the cytokine TRAIL on cell death even in the TRAIL-resistant metastatic SW620 cells. Colon carcinogenesis was initiated in Wistar rats by intraperitoneal injections of azoxymethane (AOM), once a week for two weeks. One week after (post-initiation) rats received daily Asp (0.01%, 14 mg/kg body weight) in drinking water. After 7 weeks of Asp-treatment the colon of rats exhibited a 50% reduction of the number of preneoplastic lesions (aberrant crypt foci). In addition Asp induced inhibition of several pro-inflammatory mediators, in association with an increased expression of host-defense mediators. In the colonic mucosa of Asp-treated rats we also confirmed the pro-apoptotic effects observed *in vitro* including the activation of the TRAIL death-receptor signaling pathway. Taken together, our data highlight the chemopreventive effects of Asp on colon carcinogenesis and its ability to promote normal cellular homeostasis.

## Introduction

Colorectal carcinogenesis is generally a slow process spanning decades from cancer initiation to diagnosis. Thus, this time span provides considerable opportunity to focus on the discovery and identification of dietary agents and drugs that might prevent or inhibit tumor development (1). Since about one-third of the overall risk of cancer in human may be related to diet, a large number of dietary compounds have been tested to determine their potential chemopreventive properties in various experimental cancer models (2–4). Many vegetables and their bioactive compounds exhibit strong chemopreventive ability against several neoplasms, including colorectal cancer, as evidenced by epidemiological and experimental studies (5–7). *Asparagus officinalis* L. is a popular vegetable dish consumed in most part of the world. Asparagus is rich in many bioactive phytochemicals such as steroidal saponins (8–10), flavonoids (11,12), dietary fibre (13) and oligosaccharides (14). Several asparagus constituents have been reported to possess antioxidant (12,15) anti-inflammatory (16), antitumor (10), hypolipidaemic (17) and antifungal (18) activities. However, there is a lack of information regarding the antitumor properties of asparagus constituents especially on colorectal cancer.

The aim of the present study was to gain more insight into the anti-proliferative mechanisms of a methanolic extract of white *Asparagus officinalis* shoots on human colon carcinoma cells, and to evaluate its anti-carcinogenic potential in a preclinical rat model of colon carcinogenesis. Until now, activation of cancer cell death by constituents isolated from asparagus was only shown for human promyelocytic leukemia cells (HL-60) (19), liver hepatocellular cells (HepG2) (20) and a reduction of cell viability associated with elevated expression of some apoptotic markers in colon carcinoma HCT116 cells (21).

In order to address both primary and metastatic colorectal cancer cells, we used the human colon cancer SW480 cells and their derived metastatic SW620 cells as a model for colorectal cancer progression. The SW480 cell line is isolated from a primary human colon adenocarcinoma, and the SW620 cell line is derived from the primary tumor but isolated from a mesenteric lymph node metastasis of the same patient. These two cell lines have been validated as an *in vitro* model of colon cancer progression from a primary tumor to its metastatic spreading (22).

Anticancer activity of asparagus shoots has not been demonstrated *in vivo*. In order to evaluate the chemopreventive effects of asparagus shoot extract we used the well-known azoxymethane (AOM)-induced colon cancer model in rat (23–25). In this model, one of the earliest recognizable precancerous lesions is the appearance of hyper proliferative aberrant crypt foci (ACF) about 5 weeks after cancer initiation by AOM (26–28). These foci are putative precancerous lesions that indicate the initiation of a carcinogenic process. ACF have been used as surrogate biomarkers to screen numerous potential chemopreventive agents (26), also highlighting the importance of the rat-AOM model in the screening for new drugs designed for preventive and/or therapeutic activity against colorectal cancer. Here, we extended these investigations in order to determine the effects of an oral administration of a methanolic extract of white *Asparagus officinalis* L. shoots (Asp) on the development of AOM-induced ACF formation, and on the expression of several biomarkers involved in the inflammatory and apoptotic responses in the early post-initiation phases of colon carcinogenesis.

## Materials and methods

### Plant material

The shoots of *Asparagus officinalis* L. (var. *Gijnlim*) were provided by a local asparagus producer (Spargelhof Böser, Bruchsal, Germany). The crushed shoots (about 13 kg) were freeze-dried (912 g after freeze-drying) and then extracted successively with CH_2_Cl_2_ and CH_3_OH. Extraction was performed three times with CH_2_Cl_2_ (150 ml for 15 g) for 6 h (about 25–30 extraction cycles) using a Soxhlet apparatus (150 ml, Behr, Germany). The extracts were combined and evaporated under vacuum at 40°C. The asparagus residue was then extracted three times by maceration under stirring for 2 h with CH_3_OH (150 ml for 15 g). All three methanolic extracts were then combined and concentrated under vacuum at 40°C. The crude methanolic extract (PE) was freeze-dried and subsequently purified on a C18 Sep-Pak cartridge (10 g, 35 cc, Waters, Dachstein, France). About 2 g of freeze-dried PE was dissolved in 10 ml water and loaded onto the cartridge, previously activated using 50 ml CH_3_OH + 0.1% formic acid, then conditioned with 50 ml H_2_O + 1% formic acid. The washing step was performed using 75 ml of H_2_O + 1% formic acid to remove sugars and organic acids. Fraction of interest (Asp) was then eluted from the cartridge using 75 ml of CH_3_OH + 0.1% formic acid, concentrated under vacuum and freeze-dried.

### Cell culture

SW480 and SW620 cells were obtained from the European Collection of Animals Cell Culture (Salisbury, UK). They were maintained in Dulbecco’s modified Eagle’s medium (DMEM) containing 25 mM glucose and supplemented with 10% heat-inactivated (56°C) horse serum, 100 U/ml penicillin, 100 *μ*g/ml streptomycin and 1% non-essential amino acids (Invitrogen Corp., Cergy Pontoise, France) and kept at 37°C in a humidified atmosphere with 5% CO_2_. For experiments, after trypsinization (0.5% trypsin/2.6 mM ethylenediamine tetra-acetic acid), cells were seeded at 1×10^6^ cells in culture dishes (100 mm internal diameter) or at 2×10^5^ cells in culture dishes (25 mm internal diameter). The culture medium was DMEM supplemented with 3% heat-inactivated horse serum, 100 U/ml penicillin, 100 *μ*g/ml streptomycin, 5 *μ*g/ml transferrin, 5 ng/ml selenium, 10 *μ*g/ml insulin and 1% non-essential amino acids (Invitrogen Corp.).

### Cell growth rate

Cells were exposed 24 h after seeding to different concentrations of Asp varying from 0 to 80 *μ*g/ml. The final concentration of DMSO in the culture medium was 0.1%. Control cells were treated with DMSO 0.1%. Culture medium was replaced every 48 h. At different time points, cell culture was stopped by the addition of trichloroacetic acid (50% v/v), and cell proteins were determined by staining with 200 *μ*l Sulforhodamine B (0.4% w/v) (Sigma-Aldrich, Saint Quentin Fallavier, France). The relationship between cell number (protein content per well) and absorbance is linear from 0 to 2×10^5^ cells/well.

### Cell death analysis by flow cytometry and treatments

Cells (2×10^5^) were seeded on culture dishes (25 mm diameter) and were incubated with TRAIL (50 ng/ml) (Alexis Biochemicals, Lausen, Switzerland) or with Asp (80 *μ*g/ml) or with a combination TRAIL + Asp (80 *μ*g/ml). Cells were centrifuged and fixed with 1 ml methanol: PBS (9:1 v/v), washed twice in PBS and re-suspended in 200 *μ*l PBS containing 0.25 *μ*g/ml RNase A and 0,1 mg/ml propidium iodide (Sigma-Aldrich). After incubation in the dark at 37°C for 30 min, the fluorescence of 10,000 cells was analyzed by flow cytometry and CellQuest software (FACScan, BD Biosciences, Erembodegem, Belgium).

For caspase inhibition, cells were pre-treated with the specific pan-caspase-inhibitor Z-VAD-FMK at 20 *μ*M (MBL International Corporation, Japan) for 1 h 30 min before treatment with ASP. Cells were harvested after 24 h and processed as described above.

For the study on the role of DR4 and DR5 death receptors, Human recombinant DR4/Fc and DR5/Fc chimera proteins were purchased from R&D Systems (Lille, France) and used at 100 ng/ml with a pre-treatment time of 30 min. Cells were harvested after 24 h and processed as described above.

### Measurement of caspase-3 and caspase-8 activities

Caspase activity was measured by colorimetric assay kits (Sigma-Aldrich) according to the manufacturer’s instructions. Briefly, 20 *μ*l of cell or tissue lysates were added to a buffer containing a p-nitroaniline (pNA)-conjugated substrate for caspase-3 (Ac-DEVD-pNA) or -8 (Ac-IETD-pNA) to a total reaction volume of 100 *μ*l. Incubation was carried at 37°C. The concentration of the released pNA was calculated from the absorbance values at 405 nm and the calibration curve of defined pNA solutions. Data were adjusted according to the protein content.

### Expression of TRAIL death receptors DR4 and DR5

Cells were treated with Asp (80 *μ*g/ml) and harvested by trypsinization at 24 and 48 h. Cell pellets were washed with PBS and incubated with FITC-conjugated mouse anti-human TRAIL receptors DR4 and DR5 (Alexis Biochemicals), or FITC-conjugated mouse IgG1 monoclonal isotype control antibody (BD Biosciences) for 30 min at 4°C in the dark. After washing with PBS, cells were re-suspended in PBS and the fluorescence (515 nm) of 10.000 events per sample were analyzed by FACScan and CellQuest Software (BD Biosciences).

### Real-time quantitative reverse-transcriptase polymerase chain reaction analysis

Total RNA was isolated, using the RNeasy Plus mini kit (Qiagen, Austin, TX, USA), from SW480 and SW620 cells after 24 and 48 h treatment with Asp, or from the scraped proximal colon mucosa of NaCl-injected rats, AOM-injected rats and of Asp-treated AOM-injected rats. The High Capacity cDNA Reverse Transcription kit (Applied Biosystems, Foster City, CA, USA) was used for cDNA synthesis as recommended by the supplier. RT-PCR was performed by using ABI TaqMan gene expression assays for rats: *MMP-7*, *MMP-9* (assay ID: Rn00563467; Rn00579162), *IL1β*, *TNF-α* (assay ID: Rn99999009; Rn99999017), *DEF-5*, *LCN2* (RN01478512; RN00590612) and *Bcl-2*, *Bax*, (assay ID: Rn99999125; Rn02532082), *FAS* (*CD95*), *FASL*, *DR5*, *TRAIL* (RN00685720; RN00563754; RN01753393; RN00686175); and for human SW40 and SW620 cells: *DR4* and *DR5* (assay ID: Hs00269492 and Hs00366272) according to the manufacturer’s instructions. All samples were run in triplicate in 25 *μ*l reaction volume. Quantitative real-time RT-PCR was performed by using TaqMan Universal PCR Master mix (Applied Biosystems) and the ABI Prism 7500 Sequence Detection System (Sequence detector; Applied Biosystems) in triplicate wells. The data were analyzed by a comparative threshold cycle (Ct) method. CT values were calculated using the 7500 SDS software (Applied Biosystems). The corresponding mRNA level from colonic mucosa of NaCl-injected control rats was used as an external reference. The level of *β-actin* mRNA (assay ID: Rn00667869 or assay ID: Hs99999903) of each sample was used as an internal reference to normalize the data. For the *in vivo* experiments, the fold-changes of each mRNA (mRNA relative expression) were expressed relative to the mean value of the corresponding mRNA found in the mucosa of the NaCl-injected control rats and was calculated using the 2^ΔΔCT^ method (29).

### Animals and treatments

All animal experiments were performed in accordance with the institutional guidelines of the French Ethics Committee (authorization no. A67-480, French Ministry of Agriculture). Male Wistar rats (n=24) obtained from C.E.R. Janvier (Le Genest St Isle, France) and weighing 300 g were housed under standardized conditions (22°C, 60% relative humidity, 12 h light/12 h dark cycle, 20 air changes/h) and fed a standard chow with free access to drinking water. Sixteen rats received intra-peritoneal injections of azoxymethane (AOM) (Sigma-Aldrich), at a concentration of 15 mg/kg body weight, once a week for 2 weeks. One week after the last injection of AOM (post-initiation), rats were randomly separated into two groups. One group (n=8) received daily at 5 pm a solution of 0.01% Asp (14 mg/kg body weight) in drinking water. The AOM-treated control rats (n=8) received drinking water. One group of rats (n=8) injected with 0.9% NaCl (saline) once a week for 2 weeks receiving drinking water was used as reference. Rats consumed daily about 40 ml of the drinking fluid during the whole experimental period. All animals were sacrificed 7 weeks after AOM or saline injection.

### Assessment of aberrant crypts in the colon

The determination of hyperproliferative aberrant crypts was performed on a segment of 6 cm in length, corresponding to the distal part of the colon. The segment was washed with physiological saline, cut open, pinned out flat and fixed in 10% buffered formalin. The colon was stained with 0.2% methylene blue for 5 min, rinsed in Krebs-Ringer buffer, placed onto a glass slide and examined microscopically using a low-power objective (×5) to assess hyperproliferative crypts and aberrant crypt foci (ACF). The criteria for the identification of hyperproliferative aberrant crypts were: i) increased size; ii) thicker epithelial cell lining; and iii) increased pericryptal zone relative to normal crypts. Mucosal samples of the distal colon of NaCl-injected rats, AOM-injected control rats and Asp-treated AOM-injected rats were scraped off with a glass slide and immediately frozen in liquid nitrogen for biological assays.

### Western blot analyses of protein expression

Mucosal samples were homogenized in a RIPA lysis buffer composed of 150 mM NaCl, 50 mM Tris (pH 8.0), 5 mM EDTA, using a polytron homogenizer. After ultra-centrifugation for 30 min at 10,000 × g at 4°C, the protein content was measured by the Lowry method. Equal amounts of total protein were separated by 15% sodium dodecyl sulfate (SDS)-polyacrylamide gel electrophoresis for 2 h 30 min at 65 V. Proteins were then transferred to a nitrocellulose membrane (Bio-Rad Laboratories, Marnes-la-Coquette, France). The membrane was blocked with a solution containing bovine serum albumin (BSA) 3%, Tween-20 0.1%, Tris-HCl 10 mM (pH 7.5), and 0.1% NaCl, for 1 h and, after stripping for 30 min at 50°C with a buffer containing 100 mM β-mercaptoethanol, 2% SDS and 62 mM Tris-HCl (pH 6.7), was incubated overnight at 4°C with one of the following primary monoclonal antibodies: rabbit anti-rat DR5 at 1:500 (BD Biosciences, Erembodegem, Belgium), goat anti-rat LCN2 at 1:5,000 (Calbiochem, Merck Biosciences), rabbit anti-rat MMP-7 at 1:500 (Santa Cruz Biotechnology, Santa Cruz, USA) or mouse anti-rat β-actin at 1:2,000 (Chemicon International, Hampshire, UK). The membranes were washed and incubated with 0.02 *μ*g/ml horseradish peroxidase (HRP)-conjugated goat anti-rabbit IgG (Calbiochem, Merck Biosciences) or donkey anti-goat IgG (Calbiochem, Merck Biosciences) for 1 h and visualized using the Super Signal West Pico Chemiluminescent Substrate System (Pierce).

### Statistical analysis

Data are reported as the mean ± SE. Statistical differences between control and treated groups were evaluated using the Student’s t-test or the Student-Newman-Keuls multiple comparison test.

## Results

### Effect of Asp on SW480 and SW620 cell growth

**Figure 1:**
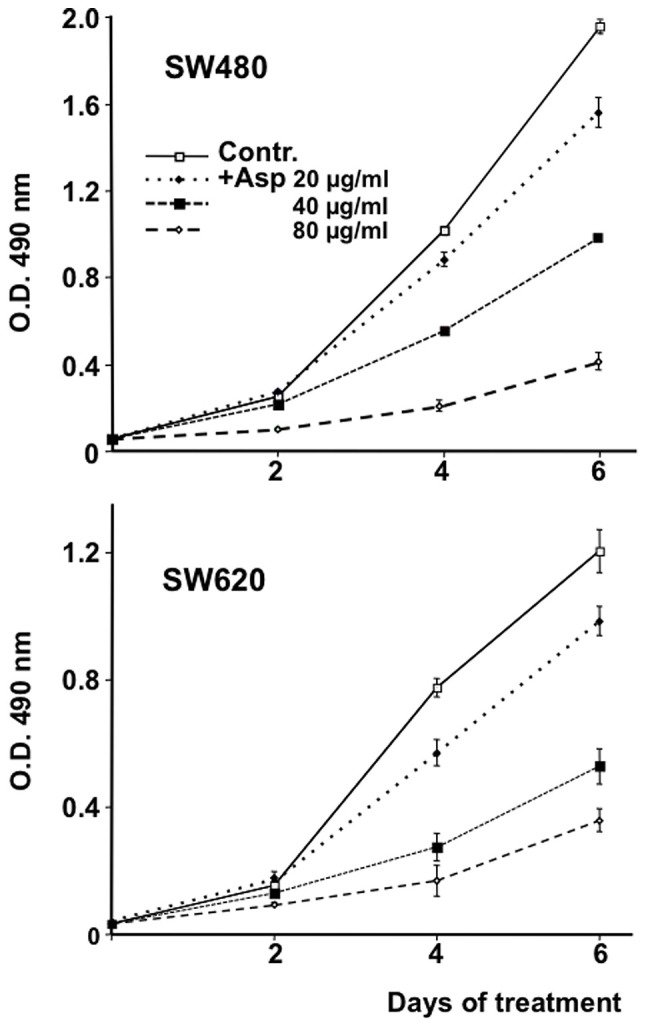
Effect of Asp on SW480 and SW620 cell growth. Cells were exposed to different concentrations of Asp varying from 0 to 80 *μ*g/ml starting 24 h after seeding. Asp was diluted in DMSO and the final concentration of DMSO did not exceed 0.1% in the culture medium. Control cells were treated with 0.1% DMSO. Data are the mean of three separate experiments.

SW480 and SW620 cells were exposed for 2 to 6 days to a methanolic extract of *Asparagus officinalis* L. shoots (Asp) at concentrations varying from 20 to 80 *μ*g/ml. As shown in Fig. 1 cell growth inhibition was observed in a dose-dependent manner. After 4 days of treatment, Asp at a concentration of 80 *μ*g/ml induced an 80% inhibition of cell growth in both cell lines.

**Figure 2:**
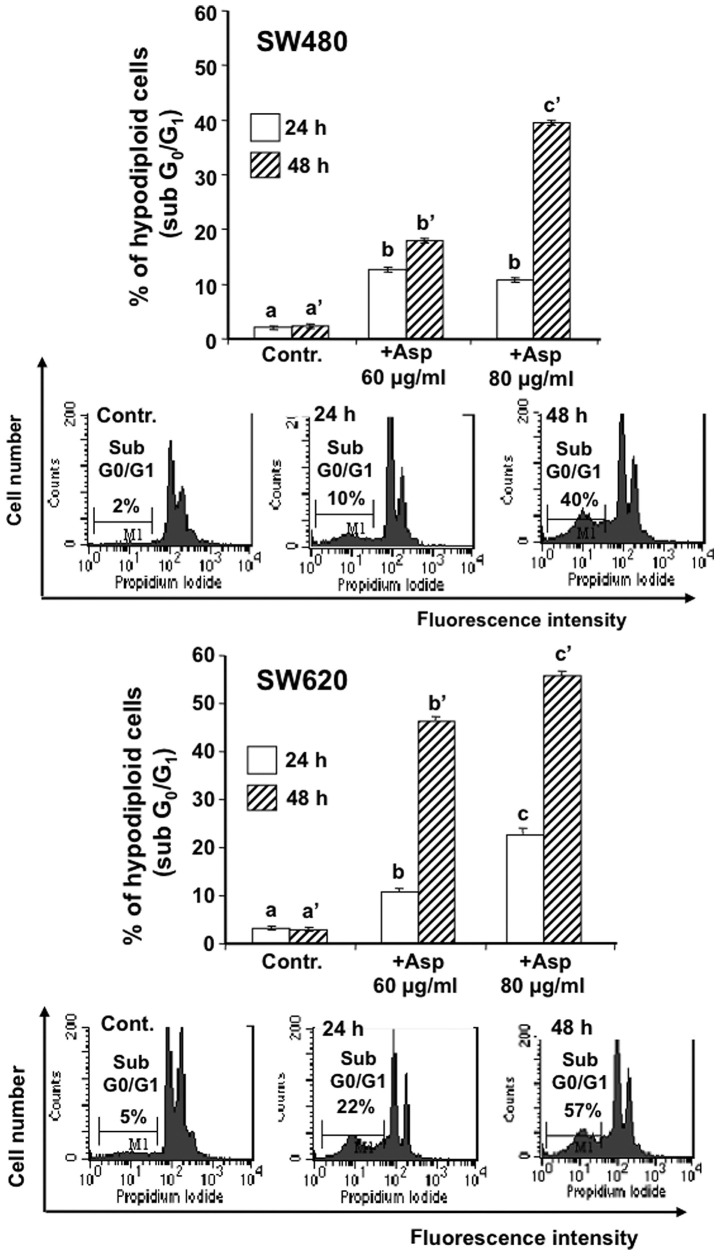
Effects of Asp on SW480 and SW620 cell death. Cells were treated with 60 or 80 *μ*g/ml Asp for 24 and 48 h. At each time point, SW480 and SW620 cells were harvested and stained with propidium iodide for the measurement of hypodiploid bodies and analyzed by flow cytometry as detailed in Materials and methods. For each cell line representative FACS histograms are shown in 48 h controls, and in SW480 and SW620 cells treated for 24 and 48 h with 80 *μ*g/ml Asp. The columns are the mean value of hypodiploid cells present in the sub G0/G1 region. At 24 or 48 h each column not sharing the same superscript letter differ significantly, P<0.05.

Propidium iodide allows the study of cell distribution in each phase of the cell cycle by measurement of cellular DNA content. After induction of cell death, DNA is degraded leading to formation of hypodiploid cells. These cells are detected by flow cytometric analysis in the sub-G0/G1 region (30). The amount of hypodiploid cells was measured 24 and 48 h after treatment with Asp. As shown in Fig. 2, the sub-G0/G1 population of SW480 and SW620 cells increased progressively with time after treatment with Asp. The percentage of hypodiploid cells was always higher in SW620 cells when compared to SW480 cells, indicating a higher sensibility of the metastatic cells to Asp treatment.

### Effects of Asp on the expression of apoptotic-related proteins

**Figure 3:**
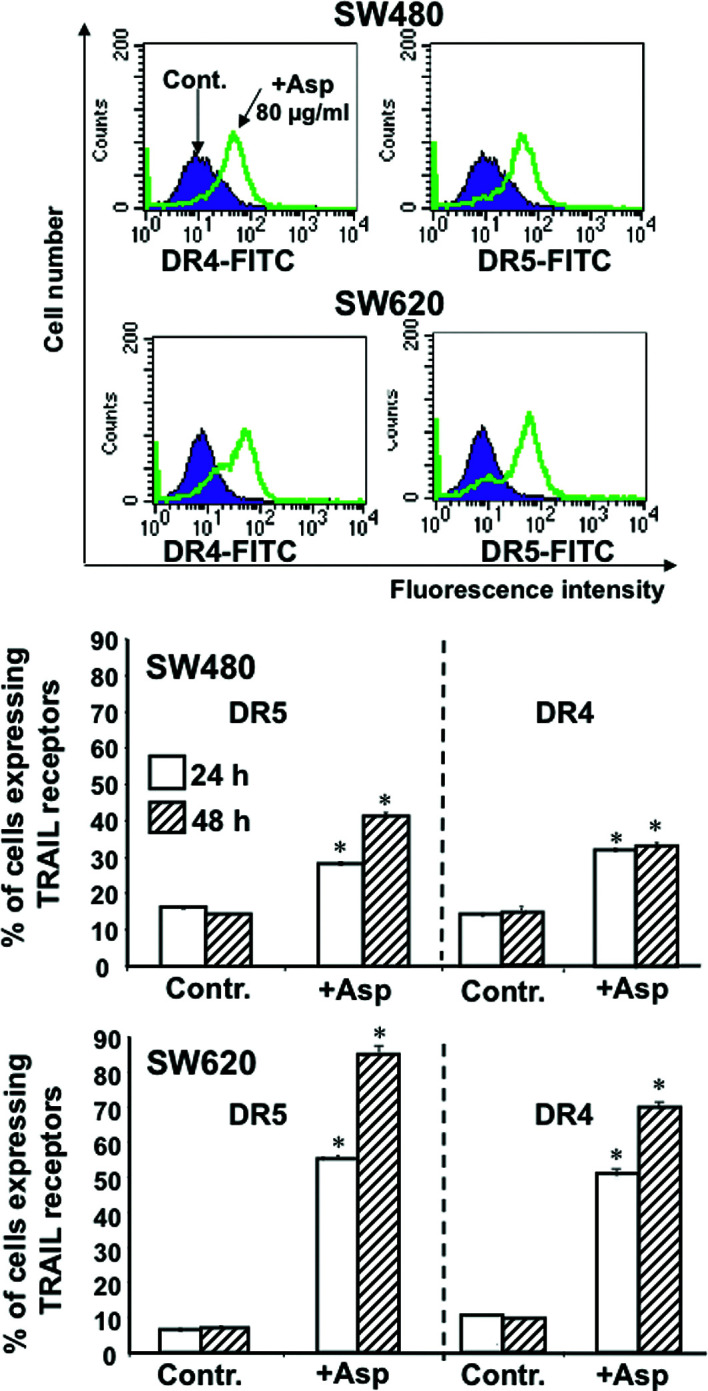
Activation of TRAIL-death receptors DR4 and DR5 by Asp. SW480 and SW620 cells were treated with Asp (80 *μ*g/ml) for 24 and 48 h. The expression of DR4 and DR5 receptors were analyzed by flow cytometry after staining with respective FITC-conjugated antibodies (details in Materials and methods). In the upper panel representative FACS histograms are shown, a shift to the right of fluorescence intensity (green line) corresponds to an increase of DR4 or DR5 receptor expression at the cell surface. In the lower panel data are presented as cytometer histogram plots representing the percentage of cells expressing DR4 or DR5 receptors. Data are the mean value ± SE of three separate experiments. For each cell line and time period: Asp vs. controls, ^*^P<0.05.

**Figure 4:**
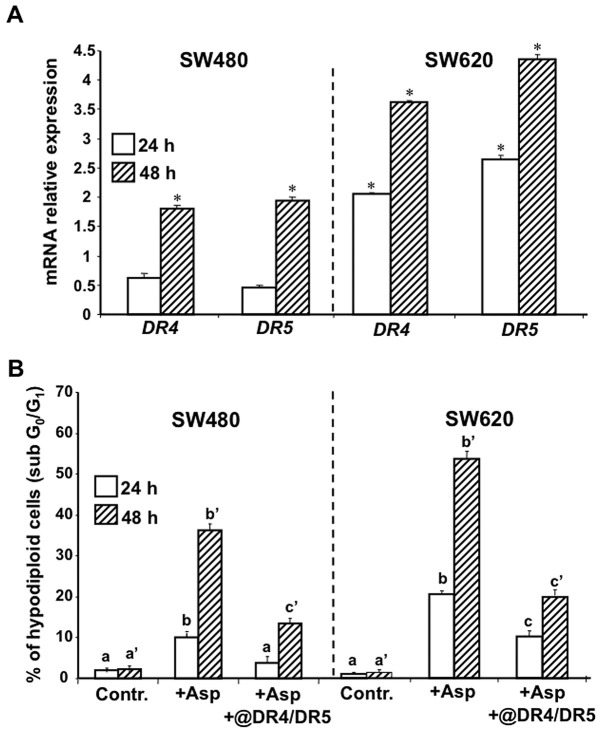
Involvement of TRAIL DR4/DR5 receptors in Asp-triggered cell death. SW480 and SW620 cells were treated with Asp (80 *μ*g/ml) for 24 and 48 h. (A) Relative expression of DR4 and DR5 mRNA expression levels in SW480 and SW620 cells. Total RNA was isolated from controls and Asp-treated cells and qRT-PCR was performed in triplicate as detailed in Materials and methods. The mRNA level from control cells was used as an external reference and the level of β-actin mRNA was used as an internal reference to normalize the data. The mRNA fold-changes (mRNA relative expression) were expressed relative to the corresponding mRNA mean value found in control cells. Data are presented as the mean ± SE. Asp vs. controls ^*^P<0.01. (B) SW480 and SW620 cells were treated with Asp (80 *μ*g/ml) ± DR4/Fc and DR5/Fc chimeric proteins (@DR4/DR5, 100 ng/ml) exhibiting a dominant-negative effect by blocking the endogenous receptors for 24 and 48 h. SW480 and SW620 cells were then harvested and stained with propidium iodide for the measurement of hypodiploid bodies and analyzed by flow cytometry as detailed in Materials and methods. Data are the mean value ± SE of at the least three separate experiments. For 24 or 48 h, columns not sharing the same superscript letter differ significantly, P<0.05.

To further explore the underlying mechanisms induced by Asp, we measured the expression of death receptor DR4 and DR5 of the TRAIL signaling pathway that tightly control apoptosis progression. We determined the protein and mRNA expression levels of DR4 and DR5 by flow cytometry and qRT-PCR. After treatment with Asp for 24 and 48 h, we observed upregulation of DR4 and DR5 protein expression at the cell surface of SW480 and SW620 cells (Fig. 3). The upregulation of DR4 and DR5 was higher in the metastatic SW620 cells when compared to the SW480 cells. Similarly, in Asp-treated SW480 cells, the amount of DR4 and DR5 transcripts remained significantly lower when compared with the level detected in SW620 cells (Fig. 4A). The DR4 and DR5 mRNA level increased significantly in SW620 cells in a time-dependent manner, respectively, by about 2-fold at 24 h and by 4-fold at 48 h compared with untreated control cells.

To demonstrate whether the observed Asp-induced cell death was mediated through the TRAIL DR4/DR5 death receptors, we used human recombinant DR4/Fc and DR5/Fc chimerical protein exhibiting a dominant-negative effect by blocking the endogenous receptors. As shown in Fig. 4B, the addition of DR4/Fc and DR5/Fc chimera (@DR4/DR5) led to a significant reduction of cell death in Asp-treated SW480 and SW620 cells.

### Caspase-8 and caspase-3 activities

**Figure 5:**
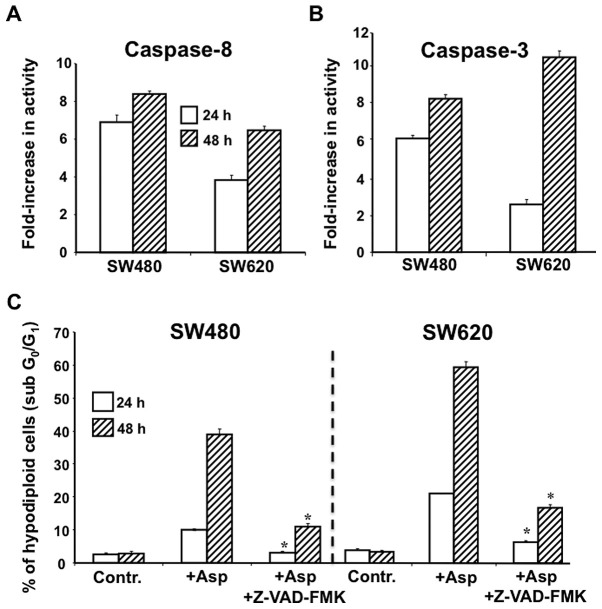
Effects of Asp treatment on caspase-8 and caspase-3 activities. Cells were treated with Asp (80 *μ*g/ml) for 24 and 48 h. The measurement of caspase activities were performed by colorimetric method using specific substrates as described in Materials and methods. Data are indicated as nmol pNA released/mg of total protein. Fold increase in activity of (A) caspase-8 and (B) caspase-3 in SW480 and SW620 cells treated with Asp. Data are presented as the mean value ± SE of at least three independent experiments. (C) Effect of caspase inhibition on Asp-induced cell death. SW480 and SW620 cells were pre-treated (for 1 h 30 min) with the pan-caspase inhibitor Z-VAD-FMK and then treated with Asp (80 *μ*g/ml) for 24 or 48 h. Cells were then harvested and stained with propidium iodide for the measurement of hypodiploid bodies and analyzed by flow cytometry. For each cell line and time-period, Asp + caspase-inhibitor vs. Asp, ^*^P<0.01.

In most cases, activation of the TRAIL death receptor pathway targets the caspase activation cascade (31). We showed that the increased expression of DR4/DR4 death receptors triggered by Asp was associated with caspase-8 (Fig. 5A) and caspase-3 (Fig. 5B) activation in both SW480 and SW620 cells. To further assess the role played by caspases in the cell death induced by Asp, we used the pan-caspase-inhibitor Z-VAD-FMK, and measured cell death after 24 and 48 h (Fig. 5C). When SW480 and SW620 cells were treated with the pan-caspase inhibitor, Asp-induced cell death was blocked.

### Sensitization of Asp-treated cancer cells to TRAIL

**Figure 6:**
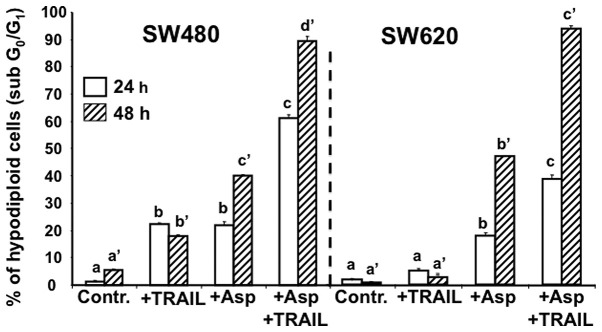
Effects of Asp and TRAIL combination on cell death. SW480 (TRAIL-sensitive) and SW620 (TRAIL-resistant) cells were treated with TRAIL (50 ng/ml) or Asp (80 *μ*g/ml) or with a combination TRAIL + Asp for 24 or 48 h. At each time point, cells were harvested and stained with propidium iodide and analysed by flow cytometry. The number of hypodiploid cells present in the subG0/G1 region is represented as histograms after 24 and 48 h of various treatments. For each cell line at 24 or 48 h, columns not sharing the same superscript letter differ significantly P<0.05.

The human colon cancer cell line SW480 is known to be TRAIL-sensitive while its derived metastatic cell line SW620 is TRAIL-resistant (32). Here, we investigated the effects of exogenous recombinant human TRAIL alone, or in combination with Asp. As shown in Fig. 6, TRAIL as a single drug exhibited only a week activity on SW480 cells and remained ineffective on SW620 cells. However, TRAIL potentiated the effects of Asp on both SW480 and SW620 cell death enhancing significantly the effects of Asp on cell death in both cell lines (95% of cell death after 48 h of treatment).

### Asp inhibits the development of preneoplastic lesions in the colon of rats

**Figure 7:**
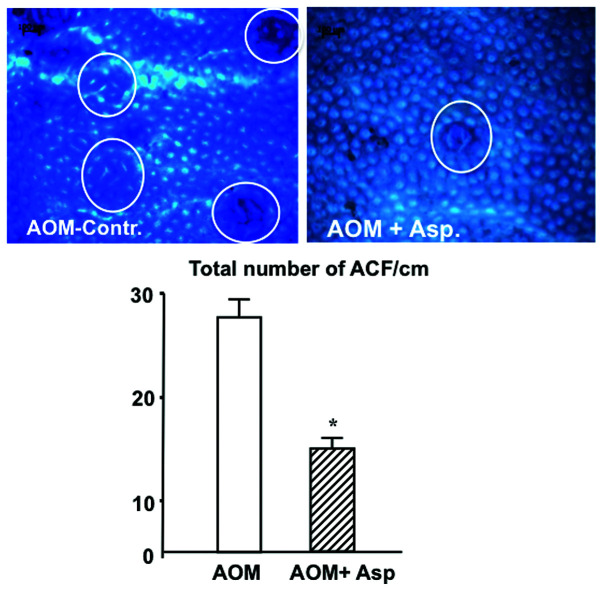
Chemopreventive effects of Asp administration in an experimental model of colon carcinogenesis. One week after the last injection of AOM (post-initiation), rats were randomly separated into two groups. One group (n=8) received daily a solution of 0.01% Asp (14 mg/kg body weight) in drinking water. The AOM-control rats (n=8) received drinking water. The upper panel shows representative views of the distal colon mucosal surface with aberrant crypt foci (in circle). Lower panel shows the total number of aberrant crypt foci (per cm length) in the distal colon in AOM-injected control rats and in Asp treated AOM-injected rats. Data are presented as the mean ± SE, ^*^P<0.01.

Based on our results showing strong anticancer effects of Asp on colon adenocarcinoma SW480 and meta-static SW620 cells in culture, we examined the efficacy of Asp in a rat model of colon carcinogenesis using azoxymethane (AOM)-initiated colon cancer. Colon carcinogenesis was induced in rats by intra-peritoneal injections of the chemical carcinogen AOM once a week for 2 weeks. Starting one week after the last injection rats received 0.01% of daily freshly prepared Asp (14 mg/kg body weight) in their drinking fluid. After 7 weeks of intervention, the colon of NaCl-injected rats exhibited no preneoplastic lesions (i.e., aberrant crypts or ACF) in contrast to the colonic mucosa of AOM-injected rats that always exhibited preneoplastic lesions. AOM-injected rats receiving Asp showed a 2-fold reduction of preneoplastic lesions when compared with the AOM control group (Fig. 7).

### Modulation by Asp of inflammatory and host-defense gene expression in colonic mucosa

**Figure 8:**
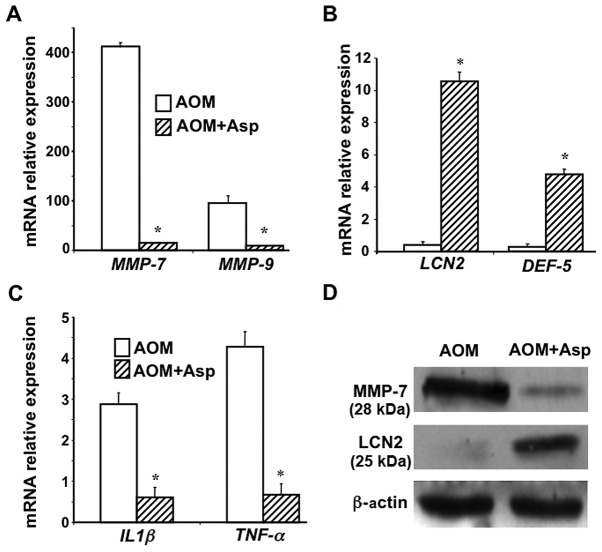
Effect of Asp treatment on the mRNA expression levels of (A) matrix metalloproteinase (*MMP-7*, *MMP-9*), (B) biomarkers of the innate immune system (*LCN2*, *DEF-5*) and (C) pro-inflammatory cytokines (*IL1β*, *TNF-α*) in the colonic mucosa of AOM-injected rats. Real-time PCR was performed in triplicate wells. The mRNA level from colonic mucosa of saline-injected control rats was used in all determinations as an external reference. The level of β-actin mRNA of each sample was used as an internal reference to normalize the data. The mRNA fold-changes (mRNA relative expression) were expressed relative to the corresponding mRNA mean value found in the colonic mucosa of saline injected rats (n=8). Data are presented as the mean ± SE, Asp treatment vs. control, ^*^P<0.01. (D) Western blot analysis of MMP-7 and LCN2 protein expression in the mucosal samples of AOM-injected control rats (AOM) and Asp-treated (+Asp) AOM-injected rats, β-actin was used as an internal control.

To gain more insight into the mechanisms underlying the *in vivo* antitumor efficacy of Asp, mucosa tissues were analyzed by quantitative real-time RT-PCR for the differential expression of inflammatory (*IL1β*, *MMP-7* and *MMP-9*) and innate immunity components (*DEF-5*, *LCN2*). In the colonic mucosa of AOM-injected rats treated with Asp, we found a significant downregulation of both *MMP-7* and *MMP-9*, close to levels measured in the mucosa of the saline-injected rats (Fig. 8A). Moreover, the amount of *MMP-7* transcript was directly correlated with the amount of MMP-7 protein (Fig. 8B). It was reported that the transcription of MMP genes is positively regulated by cytokines and growth factors such as interleukins (IL1β) or TNF-α, (33,34) both suspected to be associated with the formation of colorectal adenoma in humans (35,36). Accordingly, firstly we observed a significant upregulation of both *IL1β* (more than 2-fold) and *TNF-α* (more than 4-fold) in the colonic mucosa of AOM-injected rats compared to the expression profiles of these genes in the colonic mucosa of saline-injected rats. Secondly, the treatment of AOM-injected rats with Asp inhibited the expression of these inflammatory cytokines close to the level measured in the mucosa of saline-injected rats (Fig. 8C).

We also found that two biomarkers of the innate immune system, α-defensin-5 (*DEF-5*) and lipocalin-2 (*LCN2*), were upregulated at the mRNA level by 5- and 11-fold, respectively, in the colon of Asp-treated AOM-injected rats compared to AOM-controls or saline-injected rats (Fig. 8D). Furthermore, the levels of the *LCN2* transcripts were correlated with an increased amount of LCN2 protein (Fig. 8B). These data were consistent with the reported negative correlation between the expression levels of host-defense genes and those implicated in the inflammatory response (37).

### Asp activates apoptotic cell death in colonic mucosa

Since we observed that Asp exerted potent pro-apoptotic effects in human adenocarcinoma and metastatic cancer cells *in vitro*, we also investigated the effect of Asp on the expression of apoptotic molecules in the colonic mucosa of AOM-injected rats by RT-PCR quantitative analyses and western blot analysis.

**Figure 9:**
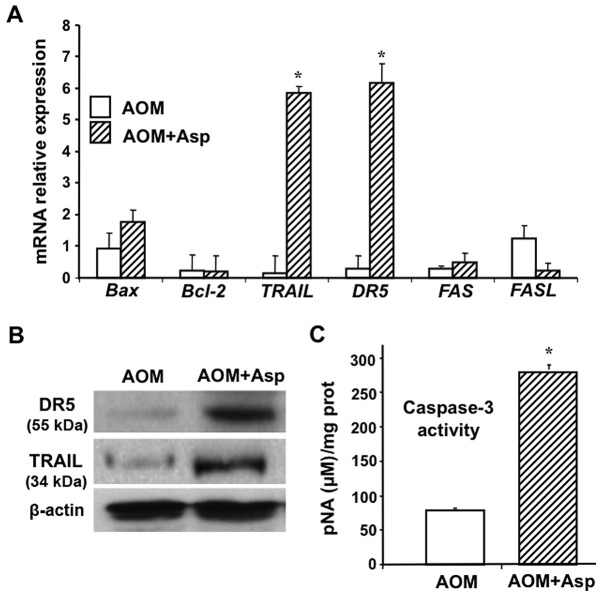
Asp activates apoptotic cell death in the colonic mucosa of AOM-injected rats. (A) Changes in the mRNA expression levels of various cell death biomarkers in the colonic mucosa of AOM-injected rats (n=8) and Asp treated AOM-injected rats (n=8). Real-time PCR was performed in triplicate wells. For further details see legend of Fig. 8. (B) Western blot analysis of the pro-apoptotic DR5 receptor and of TRAIL protein expression in the mucosal samples of AOM-injected control rats (AOM) and Asp-treated AOM-injected rats, β-actin was used as an internal control. (C) Caspase-3 activity in the colonic mucosal samples of AOM-injected control rats and Asp treated AOM-injected rats. For details see Materials and methods. Data are presented as the mean ± SE, Asp-treatment vs. control, ^*^P<0.01.

Our data showed that Asp upregulated by 6-fold the level of TRAIL-death receptor *DR5* and of *TRAIL* ligand (Fig. 9A) when compared to AOM or saline-injected rats. Furthermore, the levels of *DR5* and *TRAIL* transcripts were correlated with an increased amount of DR5 and TRAIL protein (Fig. 9B). Asp targeted specifically the TRAIL apoptotic signaling pathway since a treatment with Asp did not significantly modify the expression level of *FAS*, *FAS ligand*, *Bax* or *Bcl-2*.

In most cases, activation of the extrinsic TRAIL death receptor pathway leads to the activation of effector caspase-3. Accordingly, we found a 5-fold increase of caspase-3 activity in the mucosa of Asp-treated AOM-Injected rats when compared to AOM-injected controls (Fig. 9C). These data are in accordance with *in vitro* data showing that Asp activated caspase-dependent apoptotic signaling pathway in human colon cancer SW480 and derived metastatic SW620 cells.

## Discussion

Alterations of the processes controlling apoptosis extend the life span of cells and may favor cell neoplastic expansion independently of cell division (38). In addition, failure in apoptotic death contributes to resistance against host immune-based response and/or anticancer drug treatments. The expression of receptors belonging to the super family of tumor necrosis factor receptors such as TNF-related apoptosis inducing ligand (TRAIL) receptors DR4 and DR5, are often altered in patients with colon cancer (33). The ligand (TRAIL) by interacting with its apoptotic death receptors DR4 and DR5, selectively induces apoptosis in a wide variety of cancer cells (39). This finding has led to the development of anticancer strategies based on the use of TRAIL or TRAIL agonistic antibodies (40). However these strategies have shown only limited efficiency and the best treatment response to TRAIL agonist obtained in cancer patients from clinical studies is a stabilization of the disease (41). Nevertheless it seems that the sensitivity to TRAIL-induced apoptosis is a key factor influencing the efficacy of TRAIL treatment. Therefore, the TRAIL apoptotic pathway may be a valuable target with great potential for anticancer treatment when TRAIL or TRAIL agonists are used in combination with other anticancer therapies.

The search for dietary constituents with antitumor activity with high effectiveness on cancer cells and low toxicity on normal tissues has become one of the hotspots in research on cancer prevention and treatment (42). *Asparagus officinalis* L. is a popular vegetable in Western and Oriental countries alike. Interestingly many components of asparagus are poorly absorbed when ingested, and a substantial amount remains intact in the digestive tract where it might exert protective effects targeting the colonic mucosa. In the present study we investigated the chemopreventive mechanisms of *Asparagus officinalis* L. shoots extract (Asp), *in vitro* on human colon adenocarcinoma SW480 cells and their derived metastatic SW620 cells, and *in vivo* in a preclinical model of colon carcinogenesis in rat.

Here, we show that Asp-induced cancer cell death was mediated through the activation of the TRAIL apoptotic pathway in both adenocarcinoma SW480 cells and meta-static SW620 cells. In these cells Asp initiated a significant (P<0.01) upregulation of DR4 and DR5 transcripts and proteins. By inactivating the TRAIL death receptors with specific blocking agents (DR4/Fc and DR5/Fc) we were able to inhibit Asp-triggered cell death demonstrating the key role of TRAIL receptors in this process. It is noteworthy that Asp was able to induce the activity of both DR4 and DR5 death receptor in the metastatic SW620 cells which normally do not express active DR4/DR5 receptors at their cell surface and are known to be TRAIL-resistant (43,44). Activation of DR4/DR5 by Asp led in both cell lines to the activation of caspase-8 and caspase-3 subsequently triggering the apoptotic cell death. This was attested by the fact that pancaspase-inhibitor Z-VAD-FMK inhibited the Asp-induced cell death in these two cell lines.

From these data it can be hypothesized that Asp-treatment might represent a way to sensitize resistant cancer cells to TRAIL-treatment via the activation of DR4/DR5 death receptors. This was confirmed by our present data showing that Asp was able to potentiate the effects of TRAIL on cell death, even in TRAIL-resistant metastatic cancer cells.

Based on our *in vitro* data demonstrating the potent proapoptotic effects of Asp, we have further aimed to evaluate its chemopreventive effects in a preclinical model of colon carcinogenesis. We show that 0.01% Asp freshly administered daily in drinking water (14 mg/kg body weight) to rats, for a duration of 7 weeks starting one week after injection with a chemical carcinogen (AOM), caused a 50% reduction in the number of preneoplastic lesions (aberrant crypt foci) (ACF) at the surface of the colon. AOM-induced ACF have been widely used as a useful biomarker for colon carcinogenesis and for the evaluation of many chemopreventive agents (23,24). In order to gain more insight into the molecular targets involved in the anti-neoplastic effects of Asp, the expression of several key genes and proteins involved in the inflammatory, immune and apoptotic responses were measured in the colonic mucosa.

It is widely reported that cytokines (IL1β, TNF-α), matrix metalloproteinase (MMP-7, MMP-9) are involved in chronic inflammation, which creates a microenvironment favoring colon carcinogenesis (45). The role of these molecules have been linked to all steps involved in tumorigenesis, including initiation, cellular transformation, promotion, survival, invasion, and metastasis. Matrilysin (MMP-7) has been demonstrated to contribute to early stages of intestinal tumor progression (46). Chronic exposure to MMP-7 in premalignant epithelial cells can result in the selection of a subpopulation of cells that display a decreased sensitivity to pro-apoptotic signals. Additionally, such cells also demonstrate a significantly reduced sensitivity to drugs that stimulate apoptosis (46). Tissue inhibitors of MMPs have been suggested to be useful in combination therapy with TRAIL since it was reported that MMP inhibitors reduced tumor growth and angiogenesis in nude mice (47). Accordingly, our data indicate that *MMP-7* and, to a lesser extent *MMP-9,* are significantly enhanced in colonic mucosa of AOM-injected rats and that their expression declined significantly (P<0.01) with Asp treatment. Furthermore, we observed that Asp drastically reduced *IL1β* and TNF-α expression, which are known to induce an upregulation of MMPs (48,49). Furthermore, we observed an inverse correlation between the genetic expression of these pro-inflammatory cytokines and that of host-defense components of the innate immune system such as α-defensin-5 (*DEF-5*) and lipocalin 2 (*LCN2*), these genes being upregulated in Asp-treated AOM rats. Bioactive asparagus constituents such as saponins or fructooligosaccharides are well recognized as potent immune stimulators (50,51). These effects might be in part modulated by DEF-5 and LCN2 (38,52), which are considered as active weapons against several cancer cell types (53). Furthermore, an impaired expression of DEF-5 and LCN2 and the over-production of the pro-inflammatory cytokine IL1β have been closely associated with inflammatory bowel disease (54–56). It was also shown that DEF-5 plays an important role in the maintenance of intestinal immune homeostasis by controlling the production of IL1β (57).

Progressive inhibition of apoptosis has been described during transformation of colorectal epithelium to carcinoma. Here we showed in accordance with our *in vitro* findings that Asp-treatment of AOM-injected rats induced an important upregulation of biomarkers of the TRAIL-apoptotic pathway like DR5 and TRAIL at both gene and protein levels. These effects were also associated with an activation of caspase-3 in the colonic mucosa of Asp-treated AOM-rats.

In conclusion, this study demonstrates that a methanolic extract of white asparagus shoots activates the TRAIL death receptor pathway in the SW480 human colon adeno-carcinoma cells and in their derived TRAIL-resistant metastatic SW620 cells. This extract also sensitized these cells to TRAIL-induced apoptosis via the upregulation of death receptors (DR4/DR5) and the associated activation of caspase-8 and caspase-3, finally leading to cell death. We also demonstrated that asparagus extract administered in drinking water to AOM-injected rats exerted various anti-carcinogenic and protective effects on the colonic mucosa at early post-initiation stages. At the molecular level, the asparagus extract exhibited multi-targeted effects on the preneoplastic colonic mucosa including the inhibition of cellular pro-inflammatory mediators such as IL1β, TNF-α, MMP-7 and MMP-9, in association with an increased expression of the host-defense mediators α-defensin-5 and lipocalin 2. In the colonic mucosa of AOM-injected rats receiving the asparagus extract we confirmed the pro-apoptotic effects observed *in vitro* involving the activation of the TRAIL death receptor signaling pathway. Taken together our data highlight the chemopreventive potential of asparagus shoot extract on colon carcinogenesis and its ability to promote normal cellular homeostasis.
